# Papel de la sociedad civil en la promoción de la rendición de cuentas de los gobiernos en materia de equidad en salud en el Caribe: la Coalición Caribe Saludable^*^

**DOI:** 10.26633/RPSP.2021.104

**Published:** 2021-10-18

**Authors:** Trevor A. Hassell, Maisha T. Hutton, D. Beverley Barnett

**Affiliations:** 1 Healthy Caribbean Coalition Bridgetown Barbados Healthy Caribbean Coalition, Bridgetown, Barbados.

**Keywords:** Equidad en salud, sociedad civil, gobernanza, enfermedades no transmisibles, Región del Caribe, Health equity, civil society, governance, non-communicable diseases, Caribbean region, Equidade em saúde, sociedade civil, governança, doenças não transmissíveis, região do Caribe

## Abstract

Alcanzar la equidad en salud y abordar los determinantes sociales de la salud son aspectos fundamentales para alcanzar las metas en materia de salud y relacionadas con la salud de la Agenda para el Desarrollo Sostenible 2030 y sus Objetivos de Desarrollo Sostenible. Los marcos de referencia para la salud, como la Agenda de Salud Sostenible para las Américas 2018-2030, hacen hincapié en la reducción de las desigualdades en salud y en “no dejar a nadie atrás” en el desarrollo sostenible a nivel nacional. La equidad en salud incluye la promoción de la salud universal y el enfoque de atención primaria de salud, con un acceso equitativo de todas las personas a servicios de salud oportunos, de calidad, integrales y centrados en las personas y la comunidad que no ocasionen empobrecimiento. La rendición de cuentas por esos avances es igualmente importante, y un signo distintivo de una gobernanza adecuada. Los gobiernos tienen la responsabilidad primordial de reducir las desigualdades en salud y deben rendir cuentas de sus políticas y su desempeño. La sociedad civil es una parte interesada fundamental para promover un desarrollo nacional sostenible y equitativo, y debe formar parte de los mecanismos eficaces de rendición de cuentas. La Coalición Caribe Saludable —la única alianza regional del Caribe de organizaciones de la sociedad civil dedicada a prevenir y controlar las enfermedades no transmisibles, una prioridad de sanitaria importante acrecentada por las desigualdades— ha desempeñado un papel importante en hacer que los gobiernos rindan cuentas de la promoción de la equidad en salud. En este estudio se examinan los factores que han contribuido al éxito de la Coalición Caribe Saludable, con énfasis en la labor realizada en el marco de sus cinco pilares estratégicos —rendición de cuentas, promoción de la causa, desarrollo de capacidad, comunicación y sostenibilidad— así como los retos, las enseñanzas extraídas y otras consideraciones para lograr una mayor eficacia.

## Abstract (en)

Achieving health equity and addressing the social determinants of health are critical to attaining the health and health-related targets of the 2030 Agenda for Sustainable Development and its Sustainable Development Goals. Frameworks for health, including the Sustainable Health Agenda for the Americas 2018 – 2030, emphasize reduction of health inequities and “leaving no one behind” in national sustainable development. Health equity includes advancing universal health and the primary health care approach, with equitable access for all people to timely, quality, comprehensive, people- and community-centered services that do not cause impoverishment. Equally important, and a hallmark of good governance, is accountability for such advances. Governments have primary responsibility for reducing health inequities and must be held accountable for their policies and performance. Civil society has been recognized as a key partner in advancing sustainable and equitable national development. Effective accountability mechanisms should include civic engagement. The Healthy Caribbean Coalition (HCC), the only Caribbean regional alliance of civil society organizations working to prevent and control noncommunicable diseases—a major health priority fueled by inequities—has played a significant role in holding governments accountable for advancing health equity. This case study examines factors contributing to the success of the HCC, highlighting work under its five strategic pillars— accountability, advocacy, capacity development, communication, and sustainability—as well as challenges, lessons learned, and considerations for greater effectiveness.

## RESUMEN

Conquistar a equidade em saúde e abordar os determinantes sociais da saúde são essenciais para atingir as metas de saúde e as relacionadas à saúde da Agenda 2030 para o Desenvolvimento Sustentável e seus Objetivos de Desenvolvimento Sustentável. As estruturas para a saúde, incluindo a Agenda de Saúde Sustentável para as Américas 2018-2030, enfatizam a redução das iniquidades em saúde “sem deixar ninguém para trás”, quando se trata do desenvolvimento sustentável nacional. A equidade em saúde inclui impulsionar a saúde universal e a abordagem da atenção primária à saúde, habilitando o acesso equitativo por todas as pessoas a serviços oportunos, de qualidade, integrais, centrados no atendimento às pessoas e às comunidades de maneira a não causar o empobrecimento. A questão da responsabilidade por tais avanços é igualmente importante, e é um selo de distinção de boa gestão. Os governos são os principais responsáveis pela redução das iniquidades em saúde e precisam ser responsabilizados por suas políticas e por seu desempenho. Reconheceu-se que a sociedade civil desempenha um papel essencial na promoção do desenvolvimento nacional sustentável e equitativo. Para que sejam eficazes, os mecanismos de responsabilização devem incluir a participação cívica. A Coalizão do Caribe Saudável (HCC), a única aliança de organizações da sociedade civil que trabalha na prevenção e no controle de doenças não transmissíveis na região do Caribe — uma grande prioridade de saúde movida pelas iniquidades — tem desempenhado uma função significativa na responsabilização dos governos pelo avanço da equidade em saúde. Este estudo examina os fatores que contribuem para o sucesso da HCC e destaca o trabalho da perspectiva dos cinco pilares estratégicos — responsabilidade, promoção de causa, desenvolvimento das capacidades, comunicação e sustentabilidade —, bem como os desafios, as lições aprendidas e as considerações para que se torne ainda mais eficaz.

La Región de las Américas es una de las regiones más inequitativas del mundo (1). En el informe del 2008 de la Comisión sobre Determinantes Sociales de la Salud de la Organización Mundial de la Salud (OMS) se destacaron los numerosos estratificadores de la equidad y las repercusiones que pueden tener sobre la salud el entorno y los sistemas dentro de los cuales las personas nacen, crecen, viven, trabajan y envejecen (2). Este contexto crea una distribución desigual del poder, los recursos, las oportunidades y el estado de salud entre distintos grupos de personas (2).

## HISTORIA DE LA COALICIÓN CARIBE SALUDABLE

En diversos marcos a nivel regional y mundial (8–11) se reconoce el papel fundamental de la salud universal a la hora de proporcionar un acceso equitativo a servicios oportunos, de calidad, integrales, centrados en las personas y en la comunidad, y que no causen empobrecimiento. La salud universal incluye una estrategia de atención primaria de salud que abarca un primer nivel de atención receptivo y una red integrada de servicios de salud. Los Estados Miembros de la Organización Panamericana de la Salud (OPS), la Oficina Regional de la Organización Mundial de la Salud para las Américas, han apoyado resoluciones para avanzar hacia la salud universal y estrategias integradas para reducir las ENT.

Al igual que la prevención y el control de las ENT, la salud universal requiere un enfoque multisectorial que aborde los determinantes sociales de la salud y promueva un compromiso de toda la sociedad para fomentar la salud y el bienestar (8). Involucrar a la sociedad civil aporta múltiples beneficios relacionados con el empoderamiento, la prestación de servicios, el compromiso, la flexibilidad, la participación en la política y la credibilidad (12). Por eso, la sociedad civil es un asociado clave en materia de salud y en la rendición de cuentas conexa, donde sus funciones de “guardián” se ven materializadas.

El Caribe es una región compuesta por estados y territorios tanto insulares como continentales, de diferentes tamaños de superficie y población, así como distintos niveles de desarrollo. Esta región incluye algunos Pequeños Estados Insulares en Desarrollo, así como estados de ingresos bajos, medianos y altos según la clasificación de las instituciones financieras internacionales. Dado que es la única organización de la sociedad civil en el Caribe, la Coalición se guía por su visión de lograr que las personas del Caribe vivan felices y saludables, sin ENT crónicas, que puedan alcanzar todo su potencial y contribuir al desarrollo nacional y regional equitativo. Y esta visión está en consonancia a su vez con sus valores y principios rectores: el empoderamiento de las personas, la equidad, las asociaciones inclusivas, la transparencia y la integridad, la innovación y la rendición de cuentas (13). Esta visión es la misma para todos los países y territorios del Caribe, independientemente de sus características.

## INTERVENCIONES DE LA COALICIÓN PARA PROMOVER LA RENDICIÓN DE CUENTAS DEL GOBIERNO

Si bien la labor de la Coalición tiene muchas facetas, su papel como defensora e impulsora de la rendición de cuentas del gobierno para avanzar la equidad en la salud se pone de manifiesto en su trabajo realizado entre el 2008 y el 2018 (7), y en los materiales de información y comunicación disponibles en su sitio web (https://www.healthycaribbean.org/). A continuación, se presentan algunas de sus iniciativas, clasificadas según sus cinco pilares estratégicos: rendición de cuentas, promoción de la causa, desarrollo de capacidad, comunicación y sostenibilidad (13).

### Rendición de cuentas

Los gobiernos del Caribe deben rendir cuentas ante su población en relación con los numerosos compromisos contraídos en foros nacionales, regionales y mundiales en el campo de la salud. Un ejemplo actual es el marco de rendición de cuentas para la prevención de la obesidad infantil basado en el plan estratégico de la Coalición Caribe Saludable para el período 2017-2021 sobre prevención de la obesidad infantil en el Caribe (14). Dicho marco comprende (15):

- El mapeo y seguimiento de los compromisos políticos asumidos a nivel nacional y regional a través de la hoja de puntuación de prevención de la obesidad infantil (una base de datos en línea de políticas y programas nacionales que contribuyen a prevenir la obesidad infantil).
- El desarrollo de capacidades para las organizaciones de la sociedad civil, los jóvenes y las personas que tienen ENT mediante la concesión de subvenciones para llevar a cabo intervenciones como el establecimiento de la red de acción de las organizaciones de la sociedad civil para prevenir la obesidad infantil; seminarios en línea; reuniones regionales de partes interesadas, y el desarrollo colaborativo de materiales de comunicación y campañas en redes sociales (7).
- La participación pública como lo muestra la campaña “Too Much Junk” [demasiada comida chatarra] del 2018 y la petición en línea para una nutrición infantil más saludable, así como la plataforma digital “My Healthy Caribbean Schools” [escuelas saludables del Caribe], que da seguimiento a los entornos que conducen a la obesidad en las escuelas del Caribe (7).
- Orientación sobre la detección y gestión de conflictos de intereses, y sobre la lucha contra la interferencia de la industria a través de reuniones (16), mapeo y presentación de informes sobre la industria.
- Promoción de alto nivel por medio de cartas abiertas y privadas a los jefes de Estado y de Gobierno y a los ministros de CARICOM, en las redes sociales y al asistir a reuniones regionales de alto nivel, participar en grupos de trabajo regionales y emitir llamamientos a la acción (7), incluido el llamamiento urgente para establecer políticas de nutrición infantil saludables realizado en el 2019 (17).

Las iniciativas de rendición de cuentas sobre prevención de la obesidad infantil de la Coalición ponen de relieve un enfoque basado en los derechos, tomando como referencia la Convención de las Naciones Unidas sobre los Derechos del Niño, y la Observación General 15 del Comité de los Derechos del Niño de las Naciones Unidas (18), que indica específicamente que los Estados Miembros deben abordar la obesidad infantil.

La evaluación y la investigación son elementos clave de la rendición de cuentas, y la Coalición ha colaborado con la Universidad de las Indias Occidentales en la evaluación de la Declaración del Puerto de España de CARICOM y del impacto del impuesto sobre las bebidas azucaradas de Barbados realizada en el 2016 (7).

### Promoción de la causa

Gracias al trabajo realizado hasta la fecha, a la reputación favorable que ha adquirido, y al alto grado de interconexión social propio de la mayoría de los países del Caribe, la Coalición ha tenido acceso a los responsables de la formulación de políticas de alto nivel. La promoción de la causa que realiza la Coalición no solo aborda la rendición de cuentas, sino que también promueve acciones multisectoriales para abordar los determinantes sociales de la salud y la salud universal. La Coalición ha apoyado a una serie de “campeones” de las ENT llenos de pasión, motivación y experiencia que han participado en reuniones de alto nivel y foros internacionales; ha presentado declaraciones y documentos de posición a los responsables políticos de CARICOM (incluida la conferencia de jefes de estado y de gobierno) y a los líderes del sector público y de la sociedad civil, y se ha comunicado con los medios de comunicación nacionales, regionales e internacionales (7). En el 2015, la Coalición convocó una reunión de partes interesadas sobre el fortalecimiento de los sistemas de salud, la salud universal y las ENT (19). En el 2019, la Coalición elaboró un informe técnico sobre la salud universal (20) en preparación de la primera reunión de alto nivel de las Naciones Unidas sobre cobertura universal de salud, y lo presentó al Comité de Ministros de Salud de CARICOM en la Asamblea Mundial de la Salud del 2019.

La Coalición reconoció de forma temprana la importancia de los determinantes comerciales de la salud y la necesidad de comprometerse y colaborar con el sector privado, al tiempo que se detectan y abordan los conflictos de intereses. En el 2015, la Coalición convocó un foro del sector privado del Caribe, evaluó la contribución del sector a la reducción de las ENT y elaboró un marco para fortalecer las medidas pertinentes (21). Estos esfuerzos de colaboración con la industria buscaban fortalecer las prácticas comerciales favorables para la salud y encontrar soluciones beneficiosas para todos.

### Desarrollo de capacidades

La capacidad de los actores de la sociedad civil es imprescindible para una rendición de cuentas exitosa. El nivel de organización de las organizaciones de la sociedad civil, el número de miembros, sus habilidades técnicas y de promoción, su capacidad para movilizar y utilizar de forma efectiva los medios de comunicación, su legitimidad y representatividad, y la capacidad de respuesta y de rendición de cuentas de sus propios miembros son fundamentales para el éxito de las actividades de rendición de cuentas a nivel social. En muchos contextos, es necesario promover un entorno propicio para la sociedad civil y desarrollar la capacidad organizativa y técnica de las organizaciones de la sociedad civil (5).

El tamaño y la capacidad de las organizaciones de la sociedad civil del Caribe varía, y el desarrollo de la capacidad es una piedra angular de las actividades de la Coalición (7). La Coalición ha invitado a las organizaciones de la sociedad civil (sin costo alguno para las organizaciones) a reuniones regionales de partes interesadas y seminarios en línea, y ha otorgado subvenciones y orientación para el desarrollo y la ejecución de proyectos (7). Estas iniciativas han mejorado la capacidad de promoción, el seguimiento de las acciones gubernamentales, la comunicación, la alfabetización en materia de salud, la gestión de proyectos y la puesta en práctica de intervenciones basadas en la evidencia de las organizaciones de la sociedad civil (7). Además, al incluir a los representantes del sector público en estos ejercicios y llevar a cabo intervenciones de desarrollo de capacidades específicas para los comités multisectoriales sobre las ENT, la Coalición ha concientizado a diversos sectores sobre el impacto de sus políticas y programas sobre la salud. Estas intervenciones han puesto de relieve la importancia de los determinantes sociales de la salud, el enfoque de la salud en todas las políticas y la reducción de las inequidades en materia de salud.

La Coalición también ha hecho hincapié en el desarrollo de capacidades de las personas en situación de vulnerabilidad, incluidos los jóvenes y las personas que tienen alguna ENT. En el 2014, la Coalición creó el programa Youth4NCDs (7), cuyo objetivo es empoderar a jóvenes defensores de la causa. En el 2019, la Coalición designó asesores técnicos de los programas “Youth Voices” [voces jóvenes] y “Our Views, Our Voices” [nuestra perspectiva, nuestras voces], y ofreció capacitación sobre la promoción de la causa (https://www.healthycaribbean.org/our-views-our-voices/).

### Comunicación

Los materiales informativos y de comunicación de la Coalición, disponibles en su sitio web y en medios sociales, han sido reconocidos como un gran éxito, especialmente el informe semanal en línea *News Roundup* (7). Este método de comunicación y difusión de información es ideal para un público diverso que incluye a los miembros de las organizaciones de la sociedad civil dispersas geográficamente; sectores gubernamentales de toda la región; otros asociados nacionales, regionales e internacionales, y el público en general. Estos materiales promueven la multisectorialidad y la evidencia científica, e incluyen informes, infografías, informes técnicos dirigidos a gobiernos de CARICOM y otras partes interesadas, e informes de políticas sobre temas como el cambio climático, las ENT y los Pequeños Estados Insulares en Desarrollo, las ENT y las políticas comerciales, y las políticas nutricionales para la salud infantil.

### Sostenibilidad

La movilización de recursos es fundamental para la Coalición. Las organizaciones de la sociedad civil no pagan tasas y la Coalición no recibe subvenciones de las autoridades nacionales o regionales, por lo que depende totalmente de la financiación externa.

La Coalición es un asociado de confianza de los Ministerios de Salud y de las organizaciones intergubernamentales y no gubernamentales a nivel regional e internacional. Ha recibido apoyo de la OPS, CARICOM, el Banco de Desarrollo del Caribe, la Alianza de ENT, la Global Health Advocacy Incubator, la Fundación Mundial de Diabetes y el Programa Australiano de Ayuda Directa, entre otros (7). Las asociaciones de la Coalición con Sagicor Life Incorporated (Barbados) y CIBC/FirstCaribbean International ComTrust Foundation, dos entidades del sector privado en favor de la salud, proporcionan un apoyo continuo a la secretaría de la Coalición y ejemplifican las buenas prácticas de participación con entidades del sector privado.

En el contexto de la participación del sector privado, la orientación de la Coalición sobre conflictos de intereses representa una iniciativa pionera de rendición de cuentas dados los vínculos sociales estrechos que caracterizan a la región y la limitada movilización de recursos típica de los Pequeños Estados Insulares en Desarrollo. Al hacer énfasis en la relación entre la detección y gestión de conflictos de intereses, la rendición de cuentas y la mejora de la gobernanza, se promueve la equidad en la salud en la región.

Como líder reconocido en la prevención y el control de las ENT en el Caribe, la Coalición está representada en los grupos de expertos de la OMS, la junta de la Alianza de ENT y en las reuniones internacionales (7). Ha recibido numerosos reconocimientos (7), entre ellos el Premio Sharjah a la Excelencia en la Acción de la Sociedad Civil de ENT en el 2017 y el 2020, y el premio del Equipo de Tareas Interinstitucional de las Naciones Unidas sobre la Prevención y el Control de las Enfermedades no Transmisibles por ser un agente no estatal destacado, en el 2018.

## DISCUSIÓN

A pesar de las dificultades para determinar el impacto de su trabajo de una forma precisa debido a la naturaleza de la organización, la Coalición ha demostrado, sin lugar a duda, su valor en el Caribe (7). La Coalición ha jugado un papel crucial defendiendo la priorización de la salud en las agendas políticas y de desarrollo de la región; destacando la virtud y el valor de la rendición de cuentas; y promoviendo la necesidad de un enfoque de equidad en la salud (7).

A pesar de su papel ejemplar como defensora de la rendición de cuentas de los gobiernos para promover la equidad en la salud, la Coalición se enfrenta a varios desafíos, algunos de los cuales son consecuencia de su ubicación geográfica y de la naturaleza dispersa de las organizaciones que la conforman. Al igual que la mayoría de las organizaciones de la sociedad civil que trabajan en materia de salud en el Caribe, la Coalición se encuentra con desafíos para movilizar recursos humanos y financieros adecuados en un entorno donde los recursos son la principal limitación (7). También hay grandes desafíos a la hora de idear enfoques para fortalecer la gobernanza y las capacidades operativas de sus organizaciones, así como para lograr tener voz en los consejos de alto nivel del Caribe. Estos desafíos obstaculizan los esfuerzos de la Coalición para promover la actuación colectiva respecto a las ENT, para representar a la comunidad de las ENT y para llamar la atención sobre la necesidad de mecanismos de rendición de cuentas. Por último, la Coalición se enfrenta a la ausencia de datos desglosados para el establecimiento de líneas de base, para recabar información sobre las afirmaciones de desigualdad e inequidad, y para la medición del progreso. Si no se miden las desigualdades, no se puede adoptar un enfoque de equidad cuantificable (22).

A lo largo de doce años de experiencia, la enseñanza más importante aprendida por la Coalición Caribe Saludable es utilizar el poder de las redes y las asociaciones de forma operativa a todos los niveles del gobierno y de la sociedad. Cada vez es más evidente la importancia de la promoción de alto nivel por parte de campeones bien situados, al igual que el imperativo de escuchar, participar y promover las voces de los jóvenes y las personas que tienen alguna ENT. La validez y la necesidad de motivar al público para pedir la rendición de cuentas de los responsables de formular políticas son aspectos centrales de la filosofía de la Coalición, así como el valor estratégico de utilizar puntos de entrada eficaces, como la prevención de la obesidad infantil, para destacar las preocupaciones respecto a la equidad.

Existen muchas oportunidades para ampliar las asociaciones de la Coalición, sobre todo con el mundo académico y organismos similares. El objetivo de dichas asociaciones sería proporcionar una voz fuerte para pedir la recopilación y el desglose de datos de salud en función de los estratificadores socioeconómicos; involucrar a agentes ajenos al sector de la salud; seguir fortaleciendo a las organizaciones de la sociedad civil; continuar el desarrollo de marcos de rendición de cuentas, y reforzar la promoción y el apoyo a la multisectorialidad (todo el gobierno y toda la sociedad) con énfasis en la detección y gestión de conflictos de intereses.

## Conclusiones

El énfasis de la Coalición en la rendición de cuentas, su incesante promoción de alto nivel, el desarrollo de capacidades específico e inclusivo, los materiales informativos y de comunicación orientados al público, y la movilización de recursos innovadores y estratégicos han asegurado su reconocimiento como un agente clave en la promoción y contribución a la rendición de cuentas del gobierno para avanzar hacia la equidad en la salud. Aunque la Coalición se centra en la prevención y el control de las ENT, sus estrategias y métodos pueden ser adaptados para aplicarse a otras cuestiones de salud prioritarias en el Caribe y en otras regiones.

## Declaración.

Los autores son los únicos responsables de las opiniones expresadas en el artículo, que no necesariamente reflejan el criterio o la política de la *RPSP/PAJPH* o de la OPS.
